# Plasmalogen lipids: functional mechanism and their involvement in gastrointestinal cancer

**DOI:** 10.1186/s12944-018-0685-9

**Published:** 2018-03-07

**Authors:** Márcia Cristina Fernandes Messias, Giovana Colozza Mecatti, Denise Gonçalves Priolli, Patrícia de Oliveira Carvalho

**Affiliations:** Laboratory of Multidisciplinary Research, São Francisco University, USF, São Francisco de Assis Avenue, 218, Bragança Paulista, SP 12916-900 Brazil

**Keywords:** Plasmalogen, Cancer, Lipidomic, Biomarker

## Abstract

The plasmalogens are a class of glycerophospholipids which contain a vinyl-ether and an ester bond at the *sn-1* and *sn-2* positions, respectively, in the glycerol backbone. They constitute 10 mol% of the total mass of phospholipids in humans, mainly as membrane structure components. Plasmalogens are important for the organization and stability of lipid raft microdomains and cholesterol-rich membrane regions involved in cellular signaling. In addition to their structural roles, a subset of ether lipids are thought to function as endogenous antioxidants and emerging studies suggest that they are involved in cell differentiation and signaling pathways. Although the clinical significance of plasmalogens is linked to peroxisomal disorders, the pathophysiological roles and their possible metabolic pathways are not fully understood since they present unique structural attributes for the different tissue types. Studies suggest that changes in plasmalogen metabolism may contribute to the development of various types of cancer. Here, we review the molecular characteristics of plasmalogens in order to significantly increase our understanding of the plasmalogen molecule and its involvement in gastrointestinal cancers as well as other types of cancers.

**Electronic supplementary material:**

The online version of this article (10.1186/s12944-018-0685-9) contains supplementary material, which is available to authorized users.

## Background

Lipids, especially phospholipids (PL), are extremely diverse molecules and act as regulators of various cellular functions such as homeostasis, cell adhesion and migration, neurotransmission, signal transduction, vesicular trafficking, apoptosis and post-translational modification [1]. Among the lipids, the categories that have the most important roles in the membrane structure are glycerophospholipids (GPL), sphingolipids, sterols and triglycerides [2]. Several studies have shown that the interruption of lipid metabolism is related to the onset and severe progression of some types of human cancers [3]. Recent discoveries in the modulation of essential lipid enzymes, signaling lipid molecules and global lipid metabolism alteration in aggressive progression of cancer have fundamentally expanded our perception of lipid metabolism and its impact on tumor etiology. Rewiring of metabolic programs, such as aerobic glycolysis and increased glutamine metabolism, are crucial for cancer cells to shed from a primary tumor, overcome the nutrient and energy deficit, and eventually survive and form metastases [4].

Biomarkers can be employed for (early stage) diagnosis of cancer, prognosis (assessing lethality) and prediction 117 (of patient’s response to treatment). Some PL have been described in the literature as potential biomarkers for cancer, among them the plasmalogens, a subclass of GPL [5, 6]. Plasmalogens were discovered accidentally in 1924 by Feulgen and Voit [7] while staining sections of tissue with a nuclear dye that reacted with the aldehydes released by DNA acid hydrolysis [8]. Structurally plasmalogens exhibit a vinyl ether at the *sn-1* position of glycerol, play several roles in cellular function and are an important component of the cellular plasma membrane [9]. Although the mechanisms of action for plasmalogens remain unclear, they are starting to receive medical interest as they are now being linked to Alzheimer’s disease, Down syndrome, molecular signaling abnormalities and cancer. Plasmalogens are linearly correlated with metastases spreading in vivo*.* Therefore, they can be useful in the prognosis of the most frequently observed human cancers, particularly in pathological colorectal, breast, lung and prostate tissues.

Among the organ cancers, gastrointestinal (GI) cancers present an interesting pattern in their global distribution. GI cancers is a term for the group of cancers that affect the digestive system and includes gastric, colorectal, intestinal, hepatocellular, esophageal and pancreatic cancers [10]. According to the World Health Organization [11], in 2015 cancer caused 8.8 million deaths worldwide. Among the types of cancer deaths, the most common are lung cancer (1.69 million deaths), liver (788,000 deaths), colorectal (774,000 deaths), stomach (754,000 deaths) and breast (571,000 deaths). The incidence of cancer is increasing not only because of the limited understanding of its pathophysiology, but also because there is a restriction on access to prevention, treatment and prognosis of the disease for most patients [12]. Early detection of cancer through diagnostic, prognostic and predictive biomarkers represents a promising field of research in the identification of early stage cancer and in personalized therapies. Although recent studies have identified a few molecular biomarkers that may detect GI cancer at its early stage and progression, there is still a large gap that needs to be addressed to improve its screening, prevention and treatment [13].

Lipidomic analysis can also provide information about the nature of cell dysfunction and help identify the underlying metabolic pathways and molecular mechanisms of disease [9]. To date, analytical strategies have been applied to a wide variety of biological samples such as blood, plasma, serum, cerebrospinal fluid, urine and biological tissue derived from animal models or clinical patients [14]. Lipidomic analyses make it feasible to characterize the tumor, detect and, classify neoplastic cells and tissues and differentiate between the neoplastic and normal environment and also to evaluate the anticancer treatment (responsiveness and resistance). Consequently lipidomic analysis could lead to the discovery of new tumor biomarkers [15].

The aim of this review is to provide an overview of current knowledge of the biology and pathology of plasmalogens with an emphasis on their involvement in GI cancer. Furthermore a better understanding of plasmalogen biology in cancer could also lead to the development of better diagnostic and prognostic biomarkers or new therapeutic targets for GI cancers.

## The lipids in the human body

In the last decades there has been an intense effort to develop adequate methodologies to discover, identify and quantitatively monitor the lipids of the biological system [16]. They have several key biological functions, such as the activation of the components of the cell membranes, and they serve as a source for energy storage and participate in the autocrine and paracrine signaling pathways and autophagy [17]. This diversity of function is due to the enormous variation and complexity in the structure of their molecules since different biochemical transformations are necessary for their biosynthesis to occur [18].

The Lipid Maps [19] classification system defines the structure of lipids from two types of biochemical subunits or building blocks: ketoacyl and isoprene groups [17]. According to this approach, lipids can be classified into eight categories: fatty acids, glycerolipids, GPL, sphingolipids, saccharolipids and polyketides (derived from the condensation of ketoacyl subunits) and sterol and prenolic lipids (derived from the condensation of isoprene subunits) (Table 1) [12].

**Table 1:** Lipid Categories: Representation of the structures of the eight lipid categories [19]

Lipid Categories	Molecular structures	Representative functions
Fatty acids		Major lipid building block of complex lipids.
Glycerolipids		Membrane constituents, metabolic fuels and signaling molecules.
Glycerophospholipids		Components of the lipid bilayer of cells.
Sphingolipids		Formed by a sphingoid base backbone.
Sterol lipids		Important components of membrane lipids and function as hormones.
Prenol lipids		Function as antioxidants.
Polyketides		Commonly used antimicrobial, antiparasitic and anticancer agents.
Saccharolipids		Components of membrane lipids.

The major lipids present in the eukaryotic cell membrane are GPL, sterols and sphingolipids [20]. The major classes of GPL include: phosphatidic acid (PA), phosphatidylglycerol (PG), phosphatidylinositol (PI), cardiolipin and the aminoglycerophospholipids, namely phosphatidylcholine (PC), phosphatidylethanolamine (PE) and phosphatidylserine (PS). GPL are the most abundant in eukaryotic cells and the only subclass of GPL that presents a long chain vinyl ether in the *sn-1* position of the glycerol moiety is the plasmalogens [20, 21]. The GPL composition of membranes varies with cell type in multicellular organisms and is different in the individual organelles in eukaryotic cells. Plasmalogens are widely distributed in the biological membrane of animals and certain anaerobic bacteria [22] and appear to be associated with diverse clinical manifestations including metabolic diseases associated with oxidative stress [23] and cancer [5].

## Biomarker categories

According to the Food & Drug Administration (FDA) [24] a biological marker or a biomarker is defined as a characteristic that is measured as an indicator of normal biological processes, pathogenic processes, or responses to an exposure or therapeutic intervention.

**Fig. 1:**
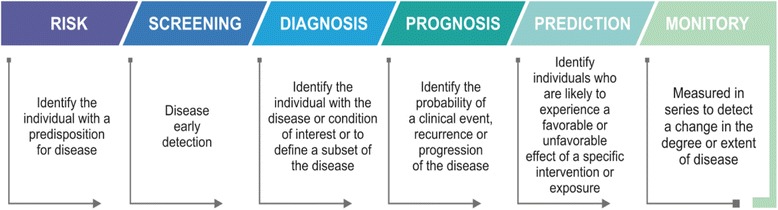
The six main biomarkers categories

They can be detected in the circulation (whole blood, serum or plasma) or excretion or secretions (stool, urine, sputum or nipple discharge), and thus easily assessed non-invasively and serially, or can be tissue–derived, and require either biopsy or special imaging for evaluation [25, 26]. In oncology, biomarkers have many potential applications inclunding risk, screening, diagnosis, prognosis, prediction and monitoring (Fig. 1) [26]. The six categories of biomarkers have been defined as follows:

- *Biomarker of risk:* inherent or acquired ability of the body to respond to exposure to a specific substance [27].
- *Biomarker screening:* early detection of disease in real time [28].
- *Biomarker diagnosis:* identifies whether a patient has a specific disease condition [29].
- *Biomarker prognosis*: informs regarding the risk of clinical outcomes such as disease recurrence or disease progression in the future [30].
- *Biomarker prediction:* predicts response to specific therapeutic interventions [25].
- *Biomarker monitory*: monitors the disease, recurrence and therapeutic response [26].

Although some approaches are performed in lipid analysis, no biomarker with 100% diagnostic accuracy has been found for any type of cancer because of the heterogeneous nature of the disease. Accordingly, efforts are focused on the search for biomarker panels instead of individual biomarkers [6]. Despite the significant improvements obtained in the last decades in the diagnosis and treatment of cancer, the impossibility of early detection of the disease through reliable biomarkers complicates personalized care for patients with cancer [1]. Reliable biomarkers could also be useful in monitoring and controlling toxicity of antitumor treatment [31].

## Characteristics of plasmalogens

Based on the substitution present at the *sn-1* position of the glycerol structure [32], GPL are divided into three subclasses: acyl, alkyl and alkenyl [33]. The alkenyls formed are called plasmalogens or plasmenyls or 1-0(1Z-alkenyl)-2-acyl- glycerophospholipids [7]. Plasmalogens are characterized by the presence of a vinyl-ether bond at the *sn-1* position and an ester bond at the*sn-2* position of the glycerol backbone [34, 35]. Other ether PL include plasmanyl PL (containing a saturated ether moiety at the *sn*-1 position), platelet-activating factor (PAF), seminolipid, and partly, the glycosylphosphatidylinositol anchor of membrane proteins. In addition to being present in human biological fluids, plasmalogens are also widely found in anaerobic bacteria, invertebrates and vertebrate animal species [7]. Plasmalogens are characterized by a short half-life: about 30 min for choline plasmalogens and 3 h for ethanolamine plasmalogens [36]. The plasmalogens are located in the cell membrane, organelles and lipid rafts and may represent (at least in selected cases) major constituents of membrane lipids; their presence is responsible for characteristic biophysical properties. The perpendicular orientation of the *sn-2* acyl chain and the lack of a carbonyl group at the *sn-1* position affect the hydrophobicity of these lipids, causing stronger intermolecular hydrogen bonding between the individual phospholipid molecules [37]. Concerning the biophysical properties, experiments have demonstrated that plasmalogens have lower lamellar gel to liquid-crystalline and lamellar to inverse-hexagonal phase transition temperatures compared to their alky and diacyl counterparts [37–39].

### General structures

**Fig. 2:**
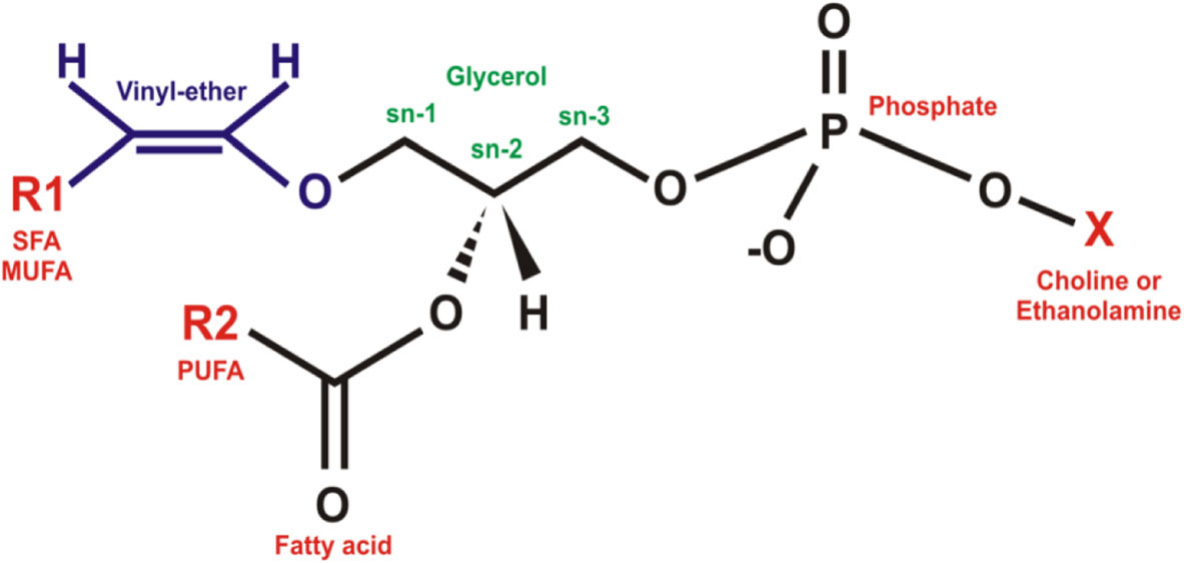
Structure of plasmalogen. R1: saturated fatty acid (SFA), monounsaturated fatty acid (MUFA); R2: polyunsaturated fatty acid (PUFA); sn-1, sn-2 and sn-3 glycerol position; X: choline or ethanolamine as polar head group

In the GPL category, plasmalogens differ from the other components of the class because they have an ether vinyl at the *sn-1* position of glycerol instead of a fatty acid [7]. To this ether vinyl (R1) are attached the saturated (C16:0) and saturated and monounsaturated carbon chains (C18:0 and C18:1, respectively) [7, 34]. In the *sn-2* (R2) position, plasmalogens are enriched with polyunsaturated fatty acid, specifically docosahexaenoic acids (C22:6 n-3) or arachidonic acid (C20:4 ω-6) [40]. As for the *sn-3* (X) position, plasmalogens are classified mainly as PC plasmalogens (also calledplasmenylcholines) and PE plasmalogens (also called plasmenylethalomines) [23] (Fig. 2).

### Distribution among different species

Plasmalogens are distributed both in the animal kingdom and in certain anaerobic microorganisms [41]. In human cells plasmalogens correspond to 10 mol% of the total mass of all PL [35]. In relation to the content of plasmalogens, human tissues and cells may differ significantly in individual values, considering that: lipoproteins contain 5% [42], myelin 11-12% [41], heart 32-50%, brain 20-50%, inflammatory cells up to 50% and spermatozoa 55% [43]. In most tissues, ethanolamine is the dominating head group. Choline plasmalogens play an important role in cardiac tissue, but represent a minor species in most other organs. Other head groups, like serine or inositol, are extremely rare. In plasma, specifically, PE and PC plasmalogens represent 50% of total PE and 5% of total PC [44]. Zhan et al. [45] point out that the liver has lower amounts of plasmalogens and that this reduction is possibly related to their synthesis in the liver and subsequent transport by the lipoproteins to other tissues.

### Biosynthesis

Peroxisomes are organelles responsible for the activity of several metabolic pathways, including plasmalogen biosynthesis and β-oxidation of long chain fatty acids [46]. The absence or dysfunction of peroxisomes may be the cause of some human diseases [40].

**Fig. 3:**
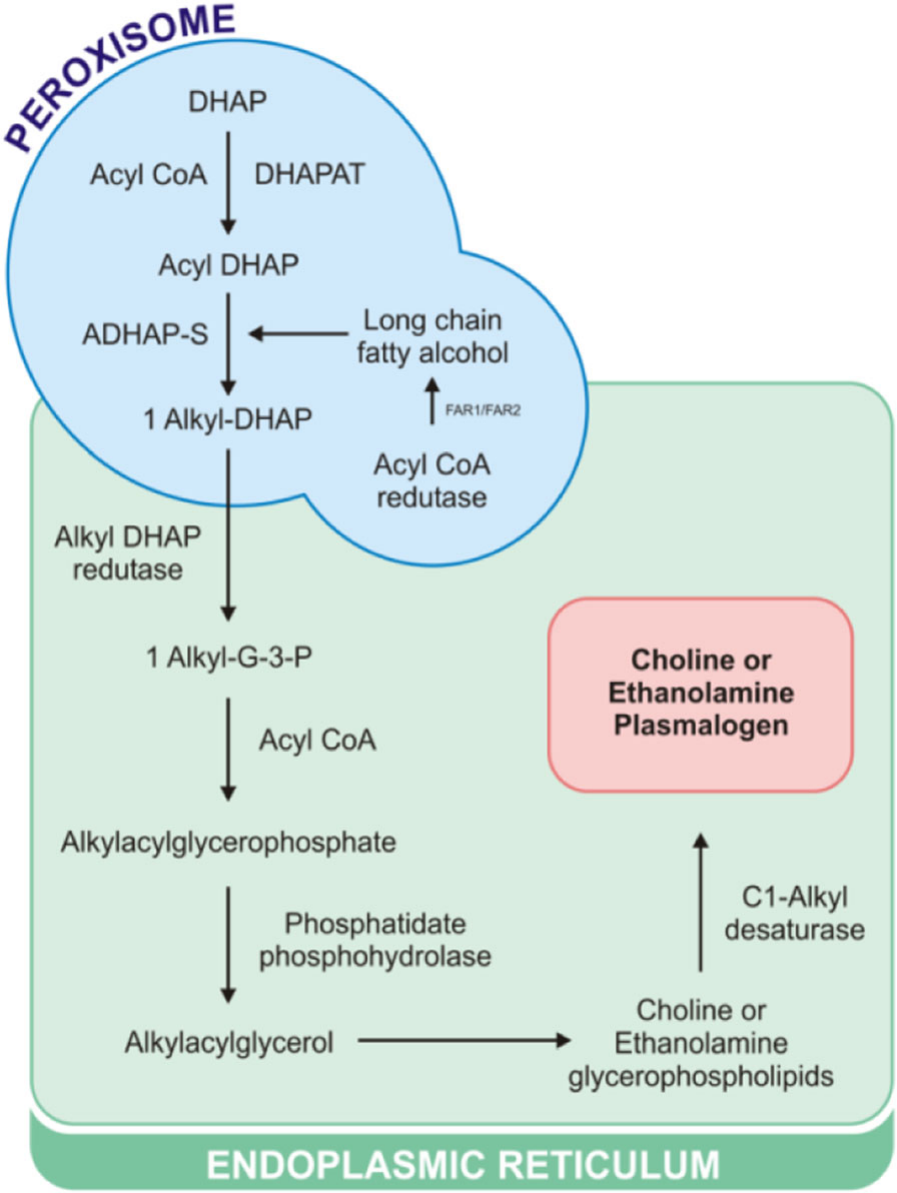
Schematic representation of the biosynthesis of plasmalogens. See text for nomenclature and abbreviations

Synthesis of plasmalogens initiated in peroxisomes occurs in seven steps (Fig. 3) and is terminated in the endoplasmic reticulum [47]. The process is initiated by the enzyme dihydroxyacetone phosphate acyltransferase (DHAPAT) where dihydroxyacetone phosphate (DHAP) is esterified with a long-chain acyl-CoA ester [48]. In the second step, the alkyl dihydroxyacetone phosphate synthase (ADHAP-S) forms the alkyl DHAP linkage. Out of the peroxisome, the acyl-CoA reductase 1 and 2 (FAR1/FAR2) enzymes provide the fatty alcohol for ADHAP-S [34]. Subsequent steps occur in the endoplasmic reticulum and there is biosynthesis of diacylglycerophospholipids [9]. In the third step alkyl-DHAP is reduced by alkyl-DHAP reductase to form 1-alkyl-glycerol-3-phosphate/G-3-P) [49]. Then, in the fourth step, 1-alkyl-G-3-P acylation occurs with acyl-CoA to produce alkylacylglycerophosphate [9]. The enzyme phosphatidate phosphohydrolase removes, in step five, the alkylacylglycerol phosphate (diacylglycerol analogue), which will be used in step six as a substrate to produce choline or ethanolamine GPL [41]. In the final step of the biosynthesis, the desaturation of the ether present in these two GPL by the C1-alkyl desaturase leads to the production of choline or ethanolamine plasmalogens [9, 41].

Membrane plasmalogen composition is tightly controlled by synthesis, remodeling, signaling induced hydrolysis and degradation. The fatty acyl-CoA reductase provides fatty alcohols used in the formation of alkyl bonds bound to ether [50]. Lysoplasmalogenase, a specific enzyme of the plasmalogens *sn-2* position, catalyzes hydrolytic cleavage of the vinyl-ether bond of lisoplasmalogen, forming a fatty aldehyde and glycerophosphocholine or glycerophosphoethanolamine [51]. It modulates the properties by similarity of the cell membrane, controlling the levels of plasmalogens and lisoplasmalogen in the cells [52]. Another enzyme that also acts in the *sn-2* position is Phospholipase A2 (PLA2). It catalyzes the hydrolysis of the *sn-2* position of glycerol, releasing arachidonic acid, a precursor of eicosanoids (prostaglandins and leukotrienes) and it also produces lysophospholipids [53].

### Functions

Although the role of plasmalogens has not yet been fully elucidated, studies suggest that they have unique functions within the cells and that these are directly related to the bonds of *sn-1* vinyl ether and *sn-2* positions of polyunsaturated fatty acids [7]. In addition, plasmalogens can act directly in reducing PL surface tension and viscosity, on the synaptic transmission process, on alveolar surfactants, improving membrane dynamics during respiratory cycles, on signal transduction [48], on membrane vesicle formation, on ion transport, on the platelet activation factor [54], the regulation of fusion, fission and fluidity of the cell membrane, control of membrane proteins activity [35], as a reservoir for second lipid messengers [41] and supporting polyunsaturated fatty acids [55].

Differences between the catabolism of ether GPL by specific phospholipase enzymes might be involved in the generation of lipid second messenger systems such as prostaglandins and arachidonic acid that are important in signal transduction [56]. Ether lipids can also act directly in cell signaling, as the PAF is an ether lipid signaling molecule that is involved in leukocyte function in the mammalian immune system [57].

Plasmalogens play a crucial role as endogenous antioxidants, protecting other PL, lipid and lipoprotein particles from oxidative stress [48]. This is due to the fact that the vinyl ether bond is preferably oxidized, while protecting the polyunsaturated fatty acids present in the *sn-2* oxidation position [55]. As the hydrogen atoms adjacent to the vinyl ether bond have relatively low dissociation energy, they end up being oxidized when exposed to various oxidizing reagents (peroxyl radicals, metal ions, UV light, singlet oxygen and halogenating species) [58]. Consequently there is consumption of plasmalogens in the reaction and the polyunsaturated fatty acids and other membrane lipids are spared from oxidation, suggesting the role of sacrificial oxidant for plasmalogens [7]. They undergo oxidative decomposition more readily than their fatty acid ester analogues [59]. The oxidative products of plasmalogens are unable to further propagate lipid peroxidation; they may terminate the lipid oxidation process [60]. Thus, it is suggested that plasmalogens interfere in the propagation step rather than in the initiation of lipid peroxidation [61]. The product profile resulting from oxidation of plasmalogens will depend on the type of fatty acid esterified in the *sn-1* and *sn-2* positions of glycerol and on the nature of oxidative stress initiators [59]. These products have been used to assess the severity of pathological conditions involving oxidative stress [61]. Plasmalogens, in addition to being prone to the oxidative process, also play a role in the inhibition of iron-induced peroxidation of polyunsaturated fatty acid and in copper-induced oxidation of low density lipoproteins [48]. Thus plasmalogens could have a decisive role in the defense systems against lipid oxidation [62].

### Analytical methods to detect plasmalogens

Several methods for identifying, characterizing and quantifying plasmalogen molecules have been developed with the aim of gaining broader knowledge about lipid ether activity in the pathogenesis of disease [35, 63]. Plasmalogen analysis can be performed through various analytical techniques (chromatography, mass spectrometry and other spectrometric techniques), each with its advantages and disadvantages [40]. Just as with any other lipid, prior to analysis by analytical methods, plasmalogens should normally be extracted using solvents such as chloroform and methanol to remove water-soluble metabolites [64]. The phase obtained with chloroform can be used without the need for purification [40]. It is worth mentioning that any addition of acid should be avoided since plasmalogens are extremely sensitive and can affect the formation of lysophospholipids and aldehydes [65].

Among the chromatographic methods used for plasmalogen analysis are Thin-layer Chromatography (TLC) and High Performance Liquid Chromatography (HPLC) [33]. These techniques, based on relative or absolute quantification and the use of internal standards, help in the identification of different plasmalogen subspecies as well as new plasmalogens [7]. TLC allows several types of samples to be investigated in a single plate [43]. In this method the plasmalogens react with acid dinitrophenylhydrazine (DNPH) leading to their hydrolysis [66]. The aldehyde released in this process is converted to 2,4-dinitrophenylhydrazone, an orange compound, which can be measured densiometrically, determining the plasmalogens content in the sample [40]. A study by Maeba & Ueta [67] with HPLC using radioactive iodine identified PC and PE plasmalogens in human plasma. Acid hydrolysis of the plasmalogens produces the lysophospholipid and a fatty acid, thus, the plasmalogen measurement is performed by quantifying one of the two products formed by HPLC [68]. Many applications of HPLC quantification for plasmalogen analysis exist and are well documented in the literature [68–70]. Currently, mass spectrometry (MS) and tandem mass spectrometry (MS/MS) methods are being used as tools for lipid analysis. Although MS has a high resolving power and mass precision, it can still be limited since many lipids have the same m/z values [71]. Strategies based on GC-MS and LC-MS for the analysis of chlorinated plasmalogen lipids (which are generated in the presence of activated chlorine) were summarized by Wacker et al. [72]. Optimized LC MS/MS conditions using alkali metals make it possible to selectively and sensitively identify PC and PE plasmalogens at the molecular species level in biological samples (rat brain and heart) [73]. Although a number of different ionization techniques are currently available in lipid research, only two of them play a major role: electrospray ionization (ESI) and matrix-assisted laser desorption and ionization (MALDI). The determination of the molecular weight alone does not provide structural information and tandem mass spectrometry (MS/MS) is normally required. Strategies currently used in lipidomics include direct infusion ESI-MS and ESI-MS/MS and MALDI combined with Fourier transform ion cyclotron resonance MS (MALDI-FTICR-MS) or time-of-flight MS (MALDI-TOF-MS) [74]. A combination of high-resolution, FI-FTICR-MS and flow-injection tandem mass spectrometry (FI-MS/MS) has been used to identify and confirm specific dysregulated metabolic systems associated with pancreatic cancer in two ethnically and geographically diverse populations [75].

A very simple method to identify plasmalogens in crude lipid extracts has been suggested. The reaction of plasmalogens with acidic dinitrophenylhydrazine (DNPH) directly leads to the hydrolysis of the plasmalogens and the subsequent conversion of the released aldehyde into a 2,4-dinitrophenylhydrazone that is easily detectable in the negative ion MALDI spectrum [66].

Individual GPL classes and even the fatty acyl composition and the linkage type in *sn-1* position of a given lipid can be differentiated by phosphorus nuclear magnetic resonance spectroscopy (^31^P NMR) [76]. Analysis of the tissue phospholipid extracts by ^31^P NMR was found to be capable of discriminating between esophageal cancer and adjacent normal tissues, including the non-involved esophagus and normal stomach [77].

## Plasmalogen lipids in gastrointestinal cancer

Decades ago it was observed that cancer cells have remarkably higher levels of alkyl and alk-1-enyl ethers lipids compared to normal cells [78–81]. Encouraged by these findings, there were efforts trying to establish ether lipids as tumor markers in medical cancer diagnostics. Some studies have also reported decreased amounts of ether lipids in cancer patients [75, 77].

In Table 2 it is possible to evaluate some plasmalogens that were identified in colorectal, gastric, pancreatic and esophageal cancer patients as well as the samples and techniques of lipidic analysis applied.

**Table 2:** Principal alkyl and alkenyl glycerolipids identified in GI cancer patients, the type of tissue sample analyzed and techniques of lipidic analysis employed

	Samples	Biomarker	Analytical Methods	References
Liver	Hepatocellular carcinoma	↑ Neutral 0-alkylglycerolipids	Gas-chromatographic (GC) and colorimetric estimation	[106]
Colon	Tissue - 16 malignant human colon and 11 non-malignant	↑ PC plasmalogen ↓ PE plasmalogen	Nuclear magnetic resonance spectroscopy (^31^P NMR)	[85]
Colon	Tissue - human colon carcinoma	↑ PC and PE plasmalogen	Thin Layer Chromatography (TLC)	[5]
Esophageal	Tissue - 36 malignant esophageal tumors	↓ PE plasmalogen	Nuclear magnetic resonance spectroscopy (^31^P NMR)	[77]
Colorectal cancer liver metastasis	Tissue – 40 liver metastases of colorectal cancers and 31 primary colorectal adenocarcinomas	↑PE plasmalogen (34:2)	Desorption electrospray ionization (DESI)	[87]
Pancreatic	Serum – 40 japanese pancreatic cancer patients and 50 controls	↓ PE plasmalogen	Flow-injection Fourier transform ion cyclotron resonance mass spectrometry (FI-FTICR-MS)	[75]
Colorectal cancer	Visceral and subcutaneous adipose tissue in 59 CRC patients (tumor stages I–IV)	↓ PC and PE plasmalogen (P-38:4/ P-36:4/) in visceral adipose tissue	Gas Chromatography time-of-flight mass spectrometry (CG-TOF-MS) and Liquid Chromatography quadrupole time-of-flight mass spectrometry (LC-QqTOF-MS)	[107]
Gastric	Plasma - 29 gastric carcinoma patients and 30 normal controls	↑ Plasmalogen	Method of Svennerholm (iodine disappearance method)	[88]
Colorectal cancer liver metastasis	Tissue - 52 liver lesions from 50 patients	↑ PE Plasmalogen (P-16:0/18:2, P-16:0/18:1)	Matrix-assisted laser desorption/ionization mass spectrometry imaging (MALDI-MSI)	[91]

Although many studies suggest altered plasmalogen production in cancer patients, the mechanism is not yet understood, suggesting a need for future research. It was observed that the plasmalogens can activate phosphatidylinositol 3-kinase, stimulate cell growth, participate in mitogenic responses [82] and have also been correlated with the levels of several oncogenic signaling lipids involved in the regulation of cell survival, cancer aggressiveness and tumor growth [83]. Benjamin et al. [83] reported increased expression of the ether lipid synthetic enzyme ADHAP-S (also called alkylglyceronephosphate synthase, AGPS) in various cancer cell lines and primary tumors. AGPS knockdown impaired experimental cancer pathogenesis, including cell survival, migration, and invasion. The pathogenic impairments conferred by AGPS knockdown in cancer cells are due to the specific depletion of the oncogenic signaling lipid lysophosphatidic acid ether and prostaglandins. The studies indicated that AGPS may serve as an attractive therapeutic target for combatting malignant human cancers, through altering the landscape of oncogenic signaling lipids that drive cancer aggressiveness [84].

Concerning the relationship of plasmalogens and tumors, Merchant et al. [85] reported a statistically significant elevation in the relative concentration of LPC and PC plasmalogens of malignant human colon specimens analyzed by ^31^P NMR. They also showed that the PE plasmalogens and PS are significantly diminished in esophageal tumors when compared to normal esophageal tissues obtained from the same patients. The data revealed a correlation between decreasing levels of four of the PL (5-dihydrosphingomyelin, lysoalkylacylphosphatidylcholine, LPC and phosphatidylglycerol) and increasing tumor aggressiveness as indicated by *T* stage and tumor grade [77].

According to Dueck et al. [5], alterations in the levels of different subclasses of plasmalogens may be related to the reduction of the activity of the Phospholipases C and D and increase of the activity of the phosphocholine cytidyltransferase enzyme in human colon cancer. For Christen et al. [86] the connection between plasmalogens and colorectal cancer can only be hypothesized, since the fluidity in the membrane, provided by plasmalogens, can facilitate the capture of carcinogenic substances, both microbial and via diet. Quantitative chromatographic analysis of the phospholipid content of colorectal carcinoma showed a generally elevated concentration of PL, also including a PE plasmalogen species (34:2) [87].

Ritchie et al. [75] confirmed the involvement of three major dysregulated metabolic systems in the serum of pancreatic cancer patients: very long-chain fatty acids, choline-containing GPL and PE plasmalogens. Although most of the individual metabolites showed a significant reduction in PC patient serum, the strongest discriminator based on multiple statistical criteria was PC-594.

In gastric carcinoma patients, the plasma plasmalogens content was significantly elevated and was positively correlated with elevated level of gangliosides and total cholesterols, but it was negatively correlated with level of total PL [88]. Although many studies consistently reported higher concentration of plasma plasmalogens in cancer patients, the mechanism is not yet understood. The mechanism may be that the key plasmalogens enzyme, phosphodihydroxyacetone acyltransferase, strengthens activity [89]. Phosphatidyl cytonucleotide transferase activity can strengthen synthesis of plasmalogen [90].

Patterson et al. [91] identify single lipid moieties that are overexpressed in different histopathological features from colorectal cancer liver metastasis specimens resected from patients preoperatively treated with chemotherapy, which have potential as new biomarkers for assessing response to therapy. Ceramides and plasmalogens were identified in the necrosis areas and made it possible to distinguish between different types of necrosis (usual necrosis and that typical of tumor progression) in tissue specimens that may not be clearly revealed by histopathology. PE plasmalogens (PE(p-16:0/18:2) and PE(p-16:0/18:1)) were associated with both tumor areas and areas of inflammation, whereas PC plasmalogens are exclusively abundant in areas of usual necrosis.

Although plasmalogens represent up to 20% of the total phospholipid mass in humans [7], found in plasma, different tissues and exosomes secreted by the colorectal cancer cell line [92], they have been excluded from profiles presented in many other studies in spite of the fact that the alterations of these active molecules are known to occur.

Altered metabolism of plasmalogens has also been reported in other cancers, such as breast, ovarian and lung cancer. Merchant et al. [85], showed that PC plasmalogens were increased in neoplastic human breast tissue compared to benign tissue and LPC was significantly depressed in benign tissue compared to normal tissue. PL indices computed to further characterize the three tissue groups showed PC plasmalogens/PC elevated in malignant tissue compared to benign tissue and PE plasmalogens/PE depressed in malignant tissue compared to noninvolved tissue. These findings support previous investigations reporting that the alkyl-phospholipid analogues of PC are released by malignant tissues and that levels of PE are elevated in malignant tissues.

Smith et al. [93] investigate the suitability of a lipid tumor marker derived from ether-linked PL in normal, benign and neoplastic samples from human breast, lung and prostate tissues. They observed that a biochemical marker derived from PE plasmalogens provides a reliable index capable of distinguishing between benign and neoplastic tissues and it correlates linearly with metastases spreading in vivo. A notable decrease of relative abundances of ether and vinylether (PIs) lipid species was detected for PEs, but no difference is apparent for PCs in tissues of breast cancer patients [94]. Recently, a lipidomics study on breast cancer patients identified increased plasma ether-linked phosphatidylcholine species as a diagnostic marker for breast cancer [95]. Compared to that found in benign patients, the plasma concentration of LPC and CE were observed to decrease in cancer patients, while PC and ether-linked phosphatidylcholine were increased. The results showed that lipid profiles may be a promising avenue for the investigation of diagnostic biomarkers of breast cancer.

Opposing trends are observed in ovarian cancer. Hou et al. [96] described that the epithelial ovarian cancer patients have reduced levels of plasmalogens compared with benign ovarian tumors and normal controls. The decreased PC and PE plasmalogens levels in these patients suggested that most cancer cells might exhibit elevated oxidative stress, which is consistent with previous findings that oxidative stress is associated with cancer progression [97].

## Anti-tumor properties of synthetic plasmalogens and analogues

Ether lipids have been shown to have anti-tumor properties, including reduction of tumor cell invasion and inhibition of tumor metastases. It is proposed that these chemotherapeutic agents interfere with lipid homeostasis due to their similarity with endogenous PL, targeting membrane lipid rafts and altering lipid-linked signalling, hence leading to apoptosis [98, 99]. Typical representatives of this group are ether PL ET-18-OCH_3_ (edelfosine) and BM 41.440 (ilmofosine) as well as hexadecylphosphocholine (miltefosine) [100]. Encouraging results have been found with all these compounds and novel, promising analogues such as erucylphosphocholine (ErPC) and its homocholine analogue erufosine (ErPC3) also hold promise as a single-agent (monotherapy) or in combination regimens [101]. Several studies combining the more recent ether lipids derivatives with diverse antineoplastic agents provide clinically significant benefits [99]. Shin et al. [102] described the synthetic pathway applied to the synthesis of 1-O-1′- (Z) -hexadecenyl-2-O-methyl-rac-glycero-3-phosphocholine, the Z-vinyl ether analogue of ET-18- OMe, which shows significant antitumor activity in pancreatic tumor cells. Also, Bittman et al. [103] reported the incorporation of a *cis*-*O*-vinyl linkage into the *sn*-1 position of glycerol of plasmalogens to synthesize a new antitumor ether lipid analogue of ET-18–OCH_3_.

The expression of plasmalogens is characteristic for tumorigenicity and an abnormal level of these glycerolipids was identified in various cancerous membranes. Flasinski et al. [104] showed that the addition of ether lipids (PAF, lyso-PAF and edelfosine) destabilizes the films on model membranes with higher choline plasmalogens content (HL-60 and normal + PC-plasma model membranes) and strengthens the interactions in systems lacking choline plasmalogens (normal leucocytes model membrane) or of lower choline plasmalogens level (K-562). It should be pointed out that cell membranes sensitive to edelfosine have a lower level of cholesterol and are enriched by choline plasmalogens (HL-60), while insensitive species have a higher sterol level and simultaneously lack plasmalogens (normal erythrocytes) or are of decreased choline plasmalogens level compared to the sensitive cells (e.g. K-562 cells). Interestingly, the level of choline plasmalogens in cells insensitive to the effect of edelfosine, normal lymphocytes and K-562 cells is lower when compared with HL-60 cells [105].

## Conclusion and perspective

Changes in plasmalogens levels were shown in this review to be exceptionally significant in biofluids and tissues of various cancer types compared with controls, which makes this category of plasmalogens good candidates as potential cancer biomarkers. This review aimed to give an overview of the current knowledge in this field with a focus on the involvement of plasmalogens in cancer. Although there is a growing body of evidence of their involvement in human diseases, many studies do not report the existence of these molecules in plasma or tissue samples from GI cancer patients. Lipidomics has just begun to enter the field of cancer diagnostics and tumor biology and the evolution of lipidomic analysis with new high-throughput, high sensitivity methods such as mass-spectrometry has made major advances in the plasmalogen field possible, by enabling detailed analyses of lipid species concentrations. Also, sophisticated statistical softwares (chemometrics) have enabled meaningful information extraction from the lipidomic data. In the near future this will lead to the identification and validation of novel, more specific biomarkers for disease detection and monitoring. The published data shows that plasmalogen levels in tissue or plasma are altered in several types of cancer. In this context, further studies should be carried out to evaluate the role of plasmalogens as potential biomarkers in patients with GI cancer and also to determine whether targeted inhibition of the vinyl-ether lipid synthetic pathway could treat these malignancies. 1

## Additional file

Additional file 1: Supplementary material. (DOCX 13 kb)

## Abbreviations

^31^P NMR: Phosphorus nuclear magnetic resonance spectroscopy
AA: Arachidonic acid
ADHAP-S: Alkyl dihydroxyacetone phosphate synthase
AGPS: Alkylglyceronephosphate synthase
DESI: Desorption electrospray ionization
DHAP: Dihydroxyacetone phosphate
DHAPAT: Dihydroxyacetone phosphate acyltransferase
DNPH: Dinitrophenylhydrazine
ESI: Electrospray ionization
FAR1/FAR2: Acyl-CoA reductase 1 and 2
FDA: Food & Drug Administration
FI-FTICR-MS: Flow-injection fourier transform ion cyclotron resonance mass spectrometry
FI-MS/MS: Flow-injection tandem mass spectrometry mass spectrometry
GC-MS: Gas chromatography–mass spectrometry
GC-TOF-MS: Gas chromatography time-of-flight mass spectrometry
GI: Gastrointestinal
GPL: Glycerophospholipids
HPLC: High performance liquid chromatography
LC-MS: Liquid chromatography–mass spectrometry
LC-QqTOF-MS: Liquid chromatography quadrupole time-of-flight mass spectrometry
LPC: Lysophosphatidylcholine
MALDI: Matrix-assisted laser desorption and ionization
MALDI-FTICR-MS: Matrix-assisted laser desorption and ionization fourier transform ion cyclotron resonance mass spectrometry
MALDI-MSI: Matrix-assisted laser desorption/ionization mass spectrometry imaging
MALDI-TOF-MS: Matrix-assisted laser desorption and ionization time-of-flight mass spectrometry
MS: Mass spectrometry
MS/MS: Tandem mass spectrometry
PA: Phosphatidic acid
PC: Phosphatidylcholine
PE: Phosphatidylethanolamine
PG: Phosphatidylglycerol
PI: Phosphatidylinositol
PL: Phospholipids
PLA2: Phospholipase A2
PS: Phosphatidylserine
TLC: Thin-layer Chromatography
