# Supplementary material for: Plasmalogen lipids: functional mechanism and their involvement in gastrointestinal cancer
Source: Lipids Health Dis. 2018 Mar 7;17:41. doi: 10.1186/s12944-018-0685-9 (PMC5842581; doi:10.1186/s12944-018-0685-9)
Supplement: Supplementary file 1 — Supplementary material. (DOCX 13 kb) [file 12944_2018_685_MOESM1_ESM.docx]

**Highlights**

Plasmalogen levels are altered in plasma and tissue samples from GI cancer patients.

Plasmalogens play roles in membrane structure, as antioxidant and as potential biomarkers of diseases.

Lipidomics analysis can be a fast, reliable and noninvasive tool to characterize the lipid profile and to monitor the progression of GI tumors
